# A DGA-aligned pulse-protein diet may support iron homeostasis in older adults: an exploratory analysis of the PRODMED1 trial

**DOI:** 10.3389/fnut.2026.1889521

**Published:** 2026-08-24

**Authors:** Bruna O. de Vargas, Jessica L. Freeling, Saba Vaezi, Moul Dey

**Affiliations:** 1 School of Health and Human Sciences, South Dakota State University, Brookings, SD, United States; 2 FreelingBio Research Consulting, LLC, Vermillion, SD, United States

**Keywords:** iron homeostasis, nutrient sensing, older adults, pork, pulses, sulfur amino acids

## Abstract

**Background/objectives:**

Iron homeostasis is essential for healthy aging, yet whether Dietary Guidelines for Americans (DGA)–aligned diets support iron status in older adults remains unclear, particularly when iron is provided exclusively as non-heme iron. This exploratory secondary analysis of PRODMED1 examined iron-related outcomes, and nutrient-responsive signals involving methionine and FGF21 in older adults consuming DGA-aligned meat- and pulse-protein diets.

**Methods:**

In an 18-week randomized crossover controlled-feeding trial, adults aged ≥60 years (*n* = 47) consumed a meat-protein diet (MPD) and a pulse-protein diet (PPD) for 8 weeks each, separated by a ≥ 2-week washout. Diets were matched for total iron but differed in heme iron exposure and sulfur amino acid content. Exploratory correlation analyses were first used to identify nutrient–iron biomarker relationships, followed by multivariable predictive analyses (MVPA) evaluating methionine and FGF21 as nutrient-responsive predictors of iron-related outcomes.

**Results:**

PPD provided iron exclusively as non-heme iron and had lower methionine content than MPD. MPD increased serum methionine, cystine, and homocysteine (all *p* ≤ 0.004), whereas FGF21 increased significantly only after PPD (*p* = 0.038). Exploratory correlations identified diet-specific nutrient–iron biomarker relationships, supporting the selection of methionine and FGF21 for MVPA. In MVPA models, ferritin was the only iron-related outcome associated with nutrient-responsive predictors; methionine (*β* = +15.2 ± 3.6, *p* ≤ 0.0001) and FGF21 (*β* = +26.2 ± 10.5, *p* = 0.012) were independently and positively associated with ferritin, with no significant associations observed for total iron-binding capacity, transferrin saturation, or soluble transferrin receptor.

**Conclusion:**

These exploratory findings suggest that a DGA-aligned pulse-protein diet may be compatible with maintained iron homeostasis in older adults despite exclusive non-heme iron intake. Methionine and FGF21 were associated with ferritin, suggesting a potential nutrient-sensing link, alongside established dietary and absorption-related determinants, both of which merit further clinical and mechanistic investigation. These findings also indicate that pulse-based protein sources may offer older adults additional shelf-stable dietary protein options, which is particularly relevant for rural communities with limited year-round access to fresh foods, without compromising iron homeostasis.

## 1 Introduction

Iron plays critical roles throughout the lifespan to support cellular functions and overall health (1, 2). Iron homeostasis is therefore particularly important for healthy aging (3), as the body’s ability to regulate iron levels can decline with age (2). Imbalances, whether through deficiency or overload, can accelerate physical and cognitive decline and increase susceptibility to infection, cardiovascular disease, and dementia (4, 5). Iron metabolism may be influenced by multiple factors such as chronic or low-grade inflammation (6, 7), changes in erythropoietic demand (8), or altered nutrient absorption (9), all of which may become more pronounced with age. These complexities create challenges for defining dietary iron needs, as some individuals are prone to deficiency (10), while others face overload (11).

Most evidence on dietary iron adequacy comes from studies using isolated nutrients, single meals, or younger populations, resulting in generalized standards (12) that fail to account for variability in aging physiology. Current recommendations emphasize total iron intake (12) but provide little guidance on how protein source, iron form, or broader dietary context affect iron regulation. Although plant sources are assumed to provide less bioavailable iron than animal sources (13–16), this may not apply within a complete dietary matrix (17). Randomized controlled trials comparing meat- and plant-based protein sources on iron status have been conducted in younger or middle-aged populations but not older adults (18, 19). Additionally, to our knowledge, there were no studies that used iron-matched, controlled feeding of Dietary Guidelines for Americans (DGA)-informed diets in any population. A more nuanced understanding of how whole-diet composition, including protein source and amino acid profile, affects iron metabolism may help refine dietary strategies for aging populations.

Absorption depends on multiple factors, including phytates, vitamin C, calcium, and iron form (heme versus non-heme). Co-ingestion of heme-rich proteins may enhance non-heme iron absorption through synergistic effects on intestinal transport (20). Amino acids themselves contribute to iron absorption, bioavailability, and homeostasis (21). Foundational work demonstrated that amino acids influence iron absorption in the gastrointestinal tract (22, 23), with more recent studies linking specific amino acid classes, like branched-chain amino acids (BCAA), to systemic iron regulation (24). Early human studies suggest that sulfur amino acids (SAA) may uniquely support iron absorption (25–27). Iron itself serves as a cofactor for many physiological functions throughout the body (28), and SAAs supply sulfur for these iron-containing cofactors (29–32). While these studies suggest that SAAs may influence iron absorption or utilization, their potential role in systemic iron regulation has not been evaluated within the context of a controlled diet matrix.

Within this context, our previously reported findings from the Protein-Distinct Macronutrient-Equivalent Diet 1 (PRODMED1) trial provided an opportunity to examine iron-related outcomes under controlled whole-diet conditions. PRODMED1 was an 18-week randomized crossover controlled-feeding study in older adults comparing DGA-aligned diets matched for total iron and macronutrient content (33). In the parent trial, both diets improved several cardiometabolic outcomes, while the pulse-based diet produced a greater increase in ferritin despite providing iron exclusively in non-heme form. In the original PRODMED1 publication, we proposed that the unexpected increase in ferritin following the pulses diet, despite pulses providing predominantly non-heme iron, may reflect greater functional responsiveness of non-heme iron within the pulse-based dietary matrix. We interpreted this in light of established differences in iron absorption, noting that non-heme iron is more sensitive to enhancers such as vitamin C and organic acids, whereas heme iron absorption from pork is more tightly regulated when iron stores are sufficient (34). This interpretation was consistent with observational and causal evidence showing that well-planned plant-forward dietary patterns can maintain or improve iron status when diet quality and absorption enhancers are adequate (35, 36).

In the present manuscript, we build on this initial interpretation by examining potential mechanistic links that could help explain the previously observed ferritin response. Rather than focusing solely on iron form, we consider whether iron homeostasis may also be influenced by the broader dietary matrix, including nutrient-sensing responses related to SAAs. We evaluate whether SAA intake, circulating methionine, and nutrient-sensitive biomarkers such as fibroblast growth factor 21 (FGF21), together with diet-specific differences in heme iron exposure, are associated with markers of iron status. Specifically, we asked whether diet-specific differences in SAA intake and circulating methionine would be associated with FGF21 and iron status biomarkers offering a nutrient-sensing explanation that complements our previous findings. In doing so, the current work extends the earlier hypothesis into a more integrated mechanistic framework, while remaining appropriately exploratory given that it draws on additional analyses of the same dataset. These analyses do not replace the previously proposed absorption-based explanation but rather extend it by considering whether systemic nutrient-sensing pathways may also contribute to the regulation of iron storage within the dietary matrix.

## 2 Methods

### 2.1 Parent trial design and participants

This study is an exploratory analysis of the PRODMED1 trial, an 18-week randomized crossover controlled-feeding study conducted in free-living older adults in Brookings, South Dakota. The parent trial design, eligibility criteria, recruitment procedures, menu development, adherence monitoring, and primary outcomes and some secondary outcomes have been reported previously (33, 37). Briefly, adults aged ≥60 years were randomized to receive two DGA-aligned dietary interventions in a crossover design: an omnivorous meat-protein diet (MPD) and a lacto-ovo vegetarian pulse-protein diet (PPD). Randomization followed a 2 × 2 block design, with blocks of two for individuals and blocks of four for couples, performed by a researcher not involved in study analyses; diet order assignment was therefore balanced across participants throughout enrollment. Given the visibly distinct nature of the two diets, full participant blinding was not feasible; blinding was maintained to the extent possible until the start of the intervention, defined as participants first dine-in meal, and all laboratory analyses of biological samples were conducted by researchers blinded to diet assignment. Each intervention phase lasted 8 weeks and was separated by a ≥2-week washout before participants crossed over to the alternate diet. The washout period was established in accordance with reported crossover nutrition studies and our prior work (38, 39) to minimize potential carryover effects; however, residual influence between dietary phases cannot be entirely ruled out. The present analysis included participants who completed both intervention phases and had available serum biomarker data (*n* = 47), including 34 women and 13 men. Analyses were conducted on a per-protocol basis; no missing data existed within the final analytic sample, and therefore no imputation was required. Physical activity, medication use, and chronic diseases that could affect iron status were assessed and comparable between randomization arms, as previously reported (33).

### 2.2 Dietary interventions and iron-related molar ratio assessment

Both intervention diets were designed as DGA-aligned healthy dietary patterns matched for total energy, macronutrient distribution, and total iron content, but differed in heme iron content and SAA composition, as previously reported (33). Briefly, the MPD used lean pork loin as the primary protein source, whereas the PPD incorporated pulses, including beans, peas, and lentils, as the primary protein source. Participants received a 7-day cyclical menu of ready-to-heat-and-eat meals providing approximately 2000 kcal/day and were instructed to consume foods *ad libitum*. Baseline dietary intake was assessed using a 24-h dietary recall. Dietary nutrient composition was analyzed using Nutritionist Pro Software (version 8.1.0, Axxya Systems), with detailed menus described in de Vargas et al. (37). SAAs (methionine and cystine) content of the diets was likewise obtained from the Nutritionist Pro database; for protein sources without available SAAs values in the software, manufacturers were contacted directly to obtain this information. Heme and non-heme iron content was estimated using total iron values obtained from Nutritionist Pro Software (based on the USDA FoodData Central), applying heme iron proportions reported by Archundia-Herrera et al. who quantified heme iron content across a range of animal-based foods (40).

Molar ratios of phytate to total iron (phytate:iron) and vitamin C to total iron (vitamin C:iron) were calculated to assess the relative dietary inhibition versus enhancement of iron absorption. Molar quantities were determined by dividing nutrient mass (mg) by molecular weight, using 660 g/mol for phytate, 176 g/mol for vitamin C, and 56 g/mol for iron. To facilitate comparison between inhibitor and enhancer effects across diets, phytate:iron and vitamin C:iron molar ratios were normalized to biologically relevant thresholds based on existing literature (34, 41–45) (phytate:iron threshold of 10 for strong inhibition; vitamin C:iron threshold of 2 for enhancement of nonheme iron absorption). A phytate “risk” score was calculated by dividing the phytate:iron molar ratio by 10, and a vitamin C “rescue” score was calculated by dividing the vitamin C:iron molar ratio by 2. An iron compensation index was then computed as the ratio of the vitamin C “rescue” score to the phytate “risk” score, serving as an empirical indicator of the relative potential for dietary components to offset inhibitory effects. This index was used as a comparative tool to contextualize dietary differences, recognizing that it is not a mechanistic or predictive model of iron absorption. Under the assumptions of this index, an iron compensation index >1 suggests adequate compensation of vitamin C over phytate.

### 2.3 Serum biomarkers

Fasting serum biomarkers were assessed at baseline and after each 8-week diet phase. Iron-related biomarkers, including serum iron, ferritin, total iron-binding capacity (TIBC), transferrin saturation (TSAT), hepcidin, erythroferrone, soluble transferrin receptor (sTfR), and the sTfR index, were measured and/or calculated as previously described (33). In the present analysis, additional biomarkers were evaluated. Insulin-like growth factor 1 (IGF-1), folate, and vitamin B12 were analyzed using the Cobas e 411 analyzer (Roche Diagnostics). Insulin-like growth factor binding protein 3 (IGFBP3) was analyzed using the MAGPIX platform with R&D Systems assays. Serum homocysteine was measured using the Centaur CP system (Siemens). All assays were performed at the University of Michigan Central Ligand Assay Satellite Services (CLASS) Laboratory.

### 2.4 Sulfur amino acid–related metabolomics

Serum samples were transported on dry ice to the NIH West Coast Metabolomics Center at the University of California, Davis, and stored at −80 °C upon receipt until analysis. Metabolites were extracted using a methanol-based protein precipitation protocol (46). Metabolomic profiling was performed using hydrophilic interaction liquid chromatography coupled to quadrupole time-of-flight mass spectrometry (HILIC-qTOFMS), incorporating isotope-labeled internal standards according to established protocols. These internal standards were used to assess extraction recovery, monitor instrument stability, and support data normalization. Absolute concentrations of cystine, methionine, and betaine were quantified using external calibration curves and reported in ng/mL. Peak detection, alignment, and metabolite annotation were performed in MS-DIAL (47), followed by manual inspection and curation of MS/MS spectra.

### 2.5 Statistical analysis and graphing

All statistical analyses were conducted in R version 4.5.0. Relationships between nutrient- and iron-related biomarkers were evaluated using a two-step analytic framework. First, exploratory Pearson correlation matrices were generated to identify pairwise associations among biomarkers, stratified by diet phase. Variables with non-normal distributions were log-transformed prior to correlation analysis. Correlated biomarker pairs were subsequently used to prioritize biologically plausible nutrient–iron relationships for multivariable modeling.

Second, multivariable predictive analyses (MVPA) were performed using linear mixed-effects models for repeated measures (LMMs) to assess associations between selected nutrient-related predictors and iron-related outcomes. Participant ID was included as a random effect to account for within-individual repeated measures, and fixed effects included timepoint, sex, nutrient predictor, and their interaction terms, including predictor × timepoint × sex interactions. FGF21 was log10-transformed prior to model entry because of its skewed distribution. All continuous predictors were mean-centered prior to model entry. Model diagnostics were evaluated for each outcome, including adjusted generalized variance inflation factors (GVIF) to assess multicollinearity and random-effect variance components, as well as residual-versus-fitted and Q–Q plots to assess residual distribution and homoscedasticity Fixed-effect estimates, standard errors, and *t*-values were extracted for each model term. Model-derived marginal slopes were estimated using the emtrends function from the *emmeans* package to quantify the adjusted change in each outcome per unit increase in the predictor, holding covariates constant. Slopes were estimated across diet phases and by sex, and slope contrasts were evaluated using the contrast function. Pairwise comparisons were adjusted using Tukey’s method within each contrast family. Given the exploratory nature of the MVPA and the number of iron outcomes and nutrient-related predictors evaluated, Benjamini–Hochberg false discovery rate (FDR) correction was additionally applied as a sensitivity analysis across the predefined family of overall methionine and FGF21 slope tests across iron-related outcomes. Corresponding 95% confidence intervals were visualized to assess group differences. For plotted associations, slope estimates and *p*-values were reported to aid interpretation. Predicted ferritin values <15 ng/mL, the World Health Organization-defined threshold for iron deficiency in adults (48), were excluded from visualizations to minimize floor effects in the lower ferritin range and improve graphical interpretability. All values were retained in model estimation to preserve the integrity of the full analytic sample and statistical inference. Because this study was conducted as an exploratory secondary analysis of the parent PRODMED1 trial, analyses were not powered *a priori* to test nutrient–iron biomarker associations, three-way interactions, or sex-stratified effects. Model complexity and stratification by sex may have reduced statistical power for some comparisons; therefore, findings were interpreted as exploratory and hypothesis-generating.

Data visualization was performed using the *ggplot2* and *pheatmap* packages in R, with final figures refined in BioRender and Adobe Illustrator. Statistical significance was defined as *p* < 0.05.

## 3 Results

### 3.1 Iron-related dietary matrix

**Figure 1:**
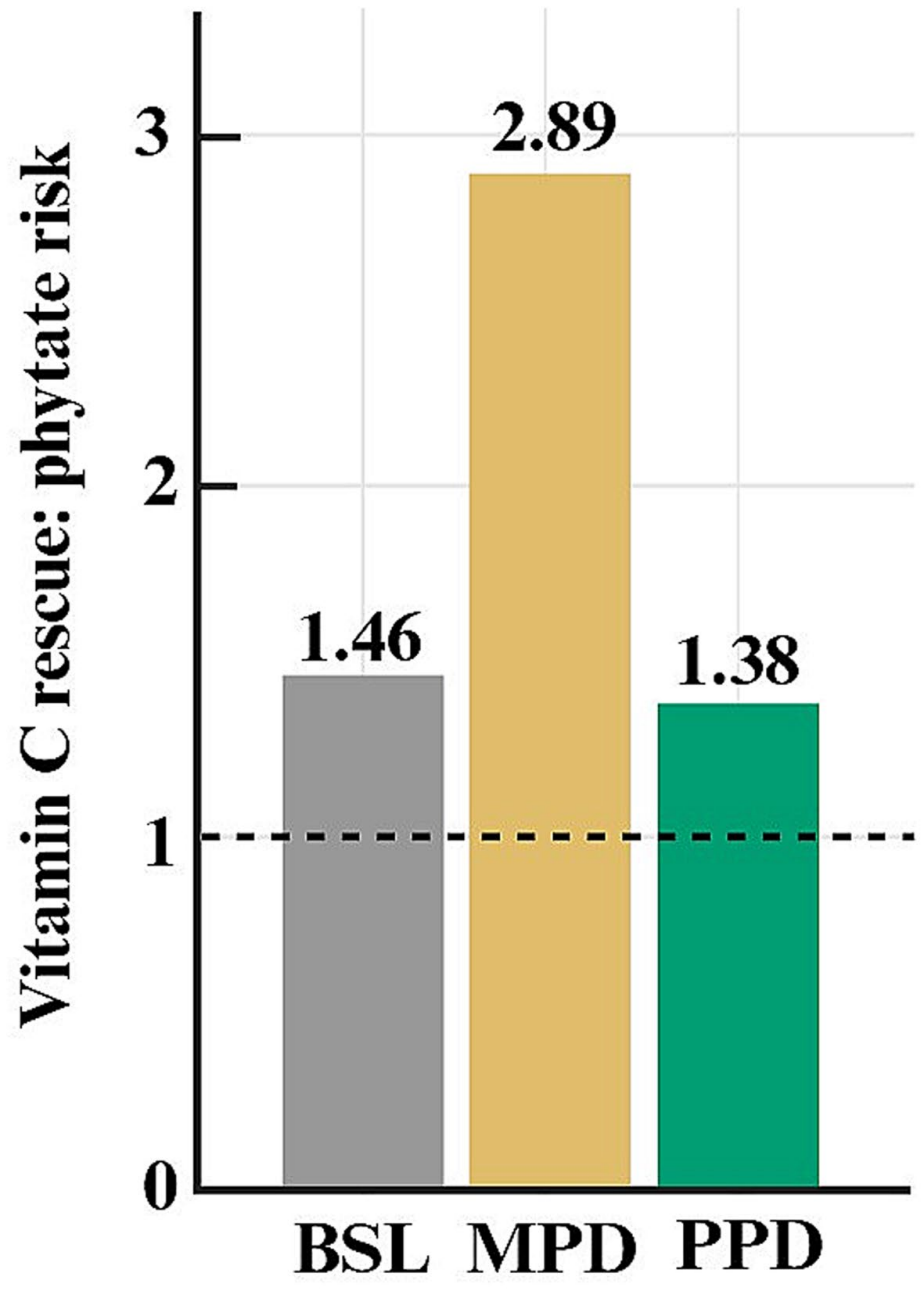
Iron compensation capacity. The iron compensation index reflects the potential of vitamin C to offset the inhibitory effects of phytate on non-heme iron absorption. BSL, baseline; MPD, meat-protein diet; PPD, pulse-protein diet.

Selected dietary components relevant to iron status are reported here; the complete dietary nutrient composition for the baseline intake and intervention diets has been described in detail elsewhere (33). The proportion of iron derived from heme sources was highest at baseline, where heme and non-heme iron contributed 1.45 mg and 12.40 mg, respectively. In MPD, heme iron accounted for 1.06 mg and non-heme iron for 17.31 mg, whereas PPD provided 18.35 mg of iron exclusively from non-heme sources. Heme iron came largely from meats and fast foods/mixed dishes at baseline and exclusively meat in MPD. Phytate levels were similar in MPD (2219.8 mg) and PPD (2457.2 mg), and approximately half those levels at baseline (1298.9 mg), corresponding to phytate:iron molar ratios of 10.25, 11.36 and 7.96, respectively. Despite the relatively higher phytate content of the intervention diets, vitamin C was above recommended dietary allowance (RDA; 75 mg and 90 mg for women and men, respectively) in all diets at 342.05 mg in MPD and 178.6 mg in PPD, with 101.4 mg reported at baseline, yielding vitamin C:iron molar ratios of 5.93, 3.10, and 2.33, respectively. The iron compensation index exceeded 1 in all dietary phases (Figure 1). The index was highest in MPD (2.89), followed by baseline (1.46) and PPD (1.38).

The intervention diets also varied in sulfur amino acid exposure. Methionine was lower in PPD (1,259 mg) than in MPD (1813 mg) and baseline intake (1,394 mg). Cystine was similar between PPD (1,024 mg) and MPD (1,045 mg), both exceeding baseline intake (785 mg). Total sulfur amino acid intake was highest in MPD (2,858 mg), followed by PPD (2,282 mg) and baseline (2,179 mg).

### 3.2 SAA metabolism and hepatic endocrine markers

MPD increased serum methionine, cystine, and homocysteine from baseline, with mean changes of +0.44 ng/mL (*p* = 0.002), +0.90 ng/mL (*p* = 0.004), and +1.8 μmol/L (*p* = 0.0004), respectively, whereas these markers did not significantly change after PPD (Table 1). Homocysteine differed between post-diet phases, with higher levels after MPD than PPD (16.9 vs. 15.4 μmol/L; *p* = 0.003). Vitamin B12 decreased from baseline after MPD (−106 pg/mL; *p* < 0.0001) but not PPD, while folate decreased from baseline after both diets (both *p* < 0.0001). Betaine increased from baseline after both MPD and PPD, with a greater post-diet value after PPD than MPD (8.12 vs. 7.35 ng/mL; *p* = 0.0005).

**Table 1:** Serum sulfur amino acid–related, one-carbon metabolism, and hepatic nutrient-sensing biomarkers.

SAA/one-carbon markers	BSL (*n* = 47)	Post MPD (*n* = 47)	*p*	Post PPD (*n* = 47)	*p*	*p*, post-post
Methionine (ng/mL)	2.85 (2.65–3.06)	3.29 (3.09–3.50)	0.002	3.16 (2.95–3.36)	0.059	0.557
Cystine (ng/mL)	4.70 (4.24–5.16)	5.60 (5.13–6.06)	0.004	5.03 (4.57–5.50)	0.470	0.112
Homocysteine (μmol/L)	15.1 (13.5–16.6)	16.9 (15.4–18.5)	0.0004	15.4 (13.8–16.9)	0.843	0.003
Vitamin B12 (pg/mL)	566 (483–650)	460 (376–543)	<0.0001	530 (447–614)	0.295	0.137
Folate (ng/mL)	18.9 (16.9–21.0)	15.4 (13.4–17.4)	<0.0001	15.3 (13.3–17.3)	<0.0001	0.991
Betaine (ng/mL)	6.29 (5.83–6.75)	7.35 (6.90–7.81)	<0.0001	8.12 (7.66–8.58)	<0.0001	0.0005
Hepatic nutrient sensing						
FGF21 (pg/mL)	189 (144–235)	223 (178–268)	0.264	242 (197–287)	0.038	0.645
IGF-1 (ng/mL)	125 (112–138)	128 (115–141)	0.779	127 (114–141)	0.800	0.999
IGFBP3 (ng/mL)	3,588 (3275–3,900)	3,133 (2820–3,445)	0.001	3,091 (2779–3,403)	0.0005	0.947

Data presented as least-squares means and 95% confidence intervals, derived from a robust linear mixed-effects model adjusted for diet phase, age, and sex, with participant ID included as a random effect. *p* values for within- and between-group comparisons were obtained from the same model.

BSL, baseline; FGF21, fibroblast growth factor 21; IGF-1, insulin-like growth factor 1; IGFBP3, insulin-like growth factor-binding protein 3; MPD, meat-protein diet; PPD, pulse-protein diet; SAA, sulfur amino acids.

FGF21 increased from baseline after PPD (+53 pg/mL; *p* = 0.038) but not MPD (+34 pg/mL; *p* = 0.264). IGF-1 remained unchanged after both diets, whereas IGFBP3 decreased from baseline after MPD (−455 ng/mL; *p* = 0.001) and PPD (−497 ng/mL; *p* = 0.0005), with no post-diet difference between MPD and PPD (Table 1).

### 3.3 Nutrient–iron biomarker correlations

**Figure 2:**
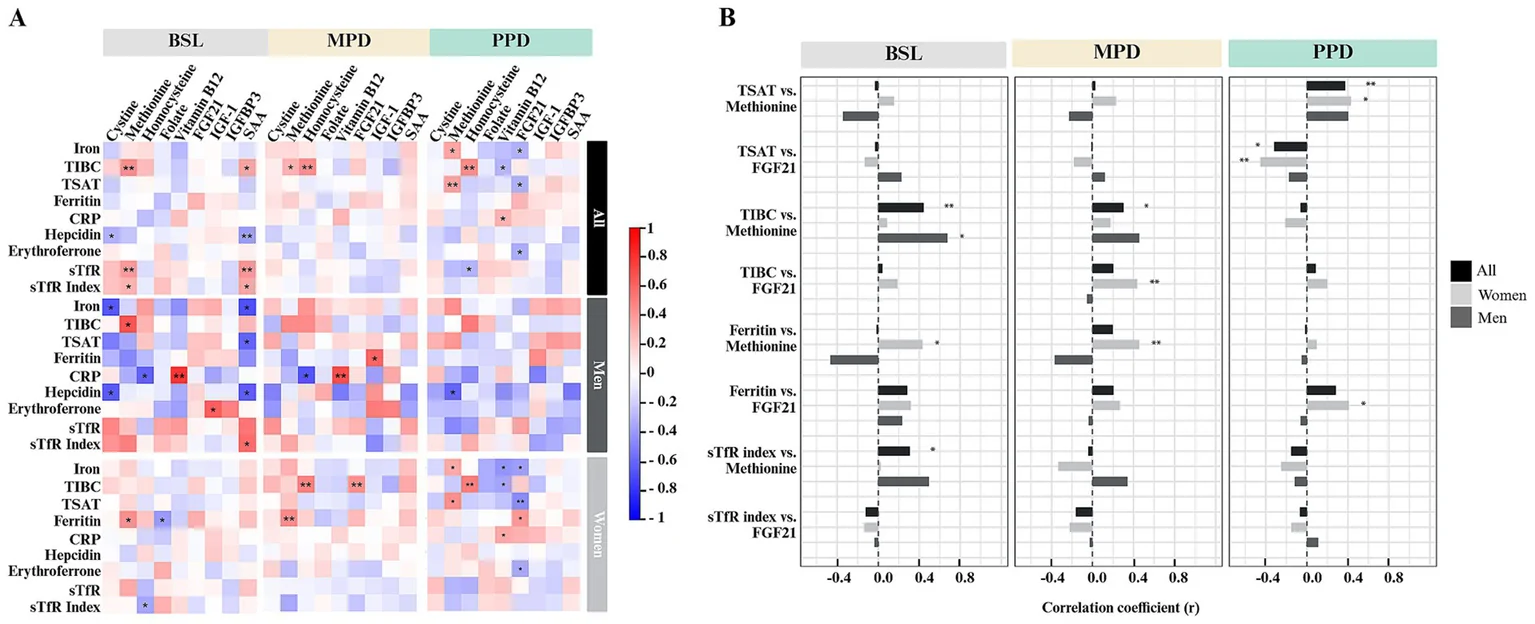
Diet-specific correlations between iron status and nutrient biomarkers. Heatmap of Pearson’s correlation coefficients between nutrient- and iron-related biomarkers, stratified by sex and diet phase **(A)**. Bar plots of selected correlations between methionine or FGF21 and iron markers **(B)**. Non-normally distributed variables were log-transformed. ^*^p < 0.05, ^**^p < 0.01. CRP, C-reactive protein; FGF21, fibroblast growth factor 21; IGF1, insulin-like growth factor 1; IGFBP3, IGF-binding protein 3; MPD, meat-protein diet; PPD, pulse-protein diet; SAA, sulfur amino acids; sTfR, soluble transferrin receptor; TIBC, total iron-binding capacity; TSAT, transferrin saturation.

Exploratory correlations between nutrient-related metabolites and iron-related biomarkers were used to identify potential nutrient–iron relationships to inform subsequent modeling (Figures 2A,B and Supplementary Table S1). A correlation matrix exploring associations by diet phase and sex is shown in Figure 2A, and selected methionine- and FGF21-related associations are summarized in Figure 2B.

Among SAA-related biomarkers, SAAs at baseline were correlated with several iron-related markers; however, closer inspection suggested that these relationships were primarily driven by serum methionine rather than cystine. Methionine showed positive associations with TIBC, TSAT, ferritin, and sTfR index, whereas comparable correlations were not observed for cystine. Notably, ferritin was positively correlated with methionine in women at baseline (*r* = 0.43, *p* = 0.01) and during MPD (*r* = 0.45, *p* = 0.007), but not during PPD. Additional support for methionine-specific relationships was provided by associations between selected iron biomarkers and methionine-related metabolites or cofactors, including homocysteine, folate, and vitamin B12. Of note, as we previously reported in the parent trial (33), ferritin increased significantly after PPD, from 65.1 to 80.8 ng/mL (*p* < 0.0001), but not after MPD, reaching 72.1 ng/mL (*p* = 0.094), with a significant post-intervention difference between diets (*p* = 0.027). Hepcidin remained unchanged after both diets, with no between-diet difference (*p* = 0.942). CRP decreased after both diets with no between-diet difference; CRP-adjustment did not alter the ferritin response pattern.

FGF21 also showed diet-specific associations with iron-related biomarkers that were not similarly observed for IGF-1 or IGFBP3. During PPD, FGF21 was inversely associated with TSAT, particularly among women (*r* = −0.45, *p* = 0.007), and was positively associated with ferritin in women (*r* = 0.41, *p* = 0.02). During MPD, FGF21 was positively associated with TIBC, again primarily among women (*r* = 0.44, *p* = 0.009). These exploratory findings supported the selection of methionine and FGF21 as nutrient-responsive candidates for subsequent multivariable modeling and suggest potential sex- and diet-specific relationships between nutrient-sensitive biomarkers and iron status.

### 3.4 MVPA of nutrient- and iron-related biomarkers

**Figure 3:**
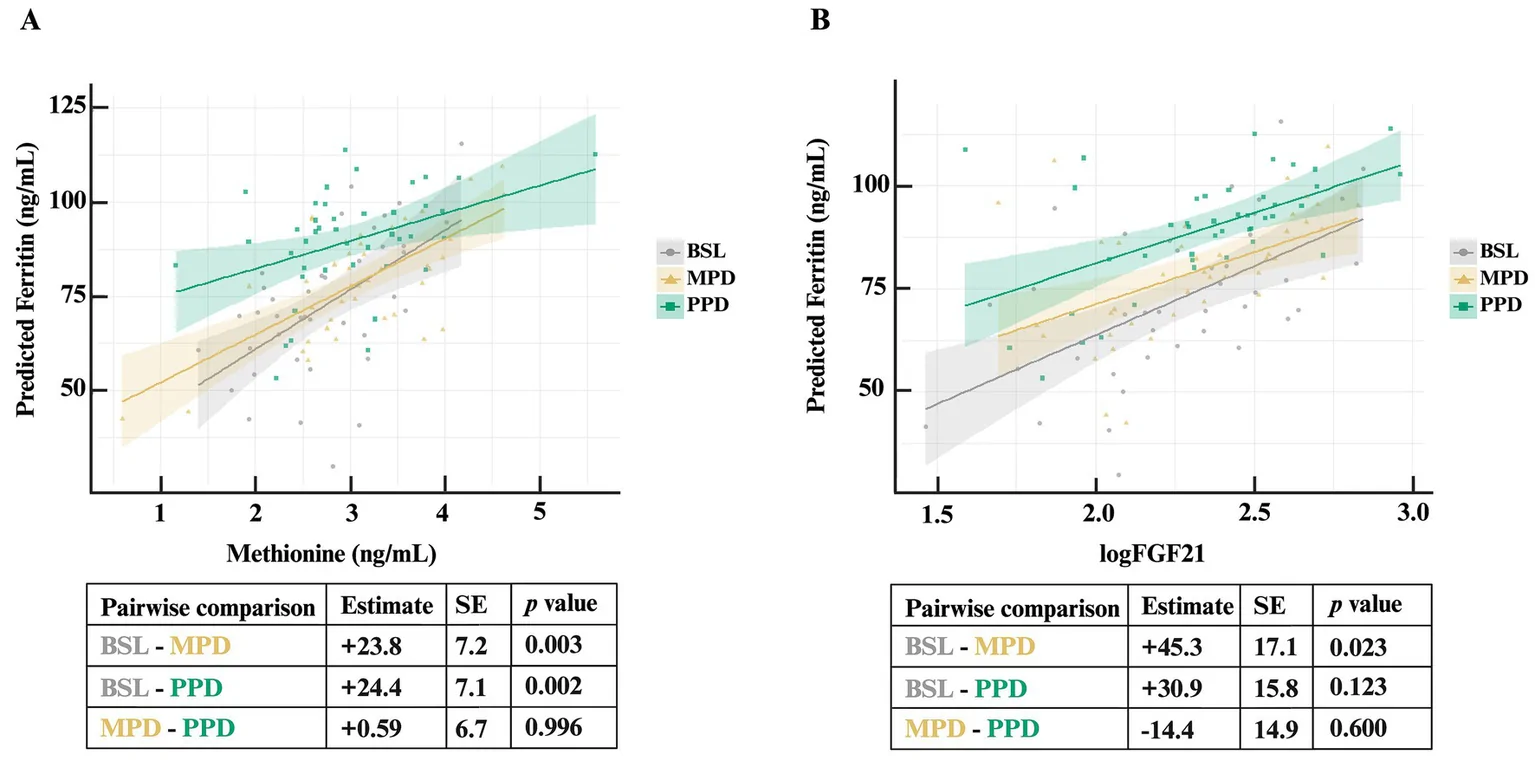
Methionine and FGF21 show study-phase-specific associations with predicted ferritin concentrations. Associations between predicted ferritin and methionine concentrations **(A)** and between predicted ferritin and log₁₀-transformed FGF21 concentrations **(B)**. Predicted ferritin values were obtained from robust linear mixed-effects models stratified by study phase. Shaded areas represent 95% confidence intervals. Slope estimates and *p*-values are shown within each panel. The tables below each plot present pairwise comparisons of slope estimates across BSL, MPD, and PPD. Observations with serum ferritin concentrations <15 ng/mL were excluded from visualization to reduce potential confounding from iron deficiency anemia but remained in the models. BSL, baseline; FGF21, fibroblast growth factor 21; MPD, meat-protein diet; PPD, pulse-protein diet; SE, standard error.

Based on the exploratory correlation patterns linking methionine- and FGF21-related signals to iron-related biomarkers, MVPA models evaluated TIBC, TSAT, ferritin, and sTfR as iron-related outcomes, with methionine and FGF21 as nutrient-responsive predictors. Full model estimates are provided in Supplementary Tables S2–S5. Among the outcomes tested, only ferritin was significantly associated with either predictor. Across all study phases, higher methionine predicted higher ferritin (*β* = 15.2 ± 3.6, 95% CI: 8.03–22.3, *p* < 0.0001), and higher FGF21 also predicted higher ferritin (*β* = 26.2 ± 10.5, 95% CI: 5.7–46.7, *p* = 0.012; Supplementary Table S2A). Both ferritin associations remained significant after FDR correction across the predefined family of overall methionine and FGF21 slope tests for iron-related outcomes (Supplementary Table S6). No significant associations were observed for TIBC, TSAT, or sTfR (all *p* ≥ 0.136; Supplementary Tables S3A–S5A), which may reflect limited statistical power, biological variability, or reduced sensitivity of these biomarkers in this exploratory analysis rather than definitive evidence of no effect. Pairwise comparisons of slopes showed that the methionine–predicted ferritin association was significantly stronger at baseline than after MPD (*β* = 23.8 ± 7.2, *p* = 0.003) or PPD (*β* = 24.4 ± 7.1, *p* = 0.002), with no difference between MPD and PPD (*β* = 0.59 ± 6.7, *p* = 0.996; Figure 3A and Supplementary Table S2B). For FGF21, the FGF21–predicted ferritin slope was significantly stronger at baseline than after MPD (*β* = 45.3 ± 17.1, *p* = 0.023), but did not significantly differ between baseline and PPD (*β* = 30.9 ± 15.8, *p* = 0.123) or between MPD and PPD (*β* = −14.4 ± 14.9, *p* = 0.600; Figure 3B and Supplementary Tables S2B). Sex-stratified slope comparisons suggested that the strength of the methionine–ferritin and FGF21–ferritin associations decreased from baseline to the intervention phases, particularly in men, with no consistent differences between MPD and PPD within sex (Supplementary Tables S2D–F, S3D–F, S4D–F, S5D–F). Overall, ferritin was the only iron-related outcome significantly predicted by methionine and FGF21 in the MVPA models, and both associations remained significant after FDR correction across the predefined family of overall slope tests. Model diagnostics, including residual-versus-fitted and Q–Q plots (Supplementary Figure S1), adjusted GVIF, and random-effect variance components (Supplementary Table S7), supported the appropriateness of the linear mixed-effects modeling approach used across all outcomes.

## 4 Discussion

This exploratory secondary analysis extends the parent PRODMED1 trial by examining iron regulation within a DGA-aligned dietary pattern differing in protein source, iron form, and SAA exposure. To our knowledge, this is among the first controlled feeding trials in older adults to show that a DGA-aligned diet with pulses as primary protein source can support iron homeostasis in older adults despite the pulse arm being fully devoid of heme-iron source. These findings challenge the common public perception that plant-based diets are inherently less effective for maintaining iron status when total diet quality, iron intake, and absorption modifiers are considered (12, 49).

PPD maintained or improved ferritin and functional iron markers, suggesting that iron status in older adults may not be explained by iron form alone. As we previously reported for this cohort (33), ferritin increased significantly after PPD but not after MPD. All additional iron-related biomarkers (TSAT, serum iron, TIBC, transferrin, sTfR, sTfR index, body iron, hepcidin, and erythroferrone) showed changes consistent with this pattern. Notably, CRP decreased significantly after both diets, with no between-diet difference, and hepcidin did not change after either diet, arguing against an inflammation-driven explanation for the observed ferritin increase. These ferritin findings remained consistent after adjustment for CRP and IL-6. Nonetheless, the sustained impact of the PPD on these clinical biomarkers require confirmation.

Both intervention diets provided high vitamin C relative to phytate exposure, and the iron compensation index exceeded 1 across all phases, consistent with evidence vitamin C’s known role in enhancing non-heme iron absorption and may counteract phytate-associated inhibition (50). Together, these results support evaluating iron nutrition within the broader dietary matrix rather than focusing only on heme versus non-heme iron. The iron compensation index, however, reflects a simplified, literature-based heuristic rather than a validated or mechanistic measure of iron bioavailability, and should be interpreted only as a tool for contextualizing relative dietary differences between diets.

A key hypothesis-generating finding was the selective association of methionine with ferritin, with FGF21 showing a weaker but complementary association. FGF21 is recognized as a hepatic nutrient-sensing hormone responsive to changes in amino acid availability, including SAA and methionine restriction (51, 52). In the present analysis, ferritin was the only iron-related outcome significantly associated with both methionine and FGF21, whereas TIBC, TSAT, and sTfR were not associated with either predictor. However, the methionine–ferritin association was more consistent across models than the FGF21–ferritin association, suggesting that methionine-related nutrient signals may be more closely linked to iron storage than to circulating iron availability, transport, or cellular iron demand.

The methionine–ferritin association was observed across baseline, MPD, and PPD and was strongest at baseline, with significant attenuation after both intervention diets and no meaningful difference between MPD and PPD slopes. Sex-stratified analyses suggested that this attenuation was more pronounced in men, whereas women showed more stable slope patterns across dietary phases. This suggests that the relationship was not specific to animal- or plant-based protein source alone but may reflect differences between habitual baseline intake and controlled, DGA-aligned dietary patterns, with possible sex-specific variation in response strength. However, these sex-specific patterns should be considered exploratory and require confirmation in adequately powered studies before firm conclusions can be drawn. For FGF21, the ferritin slope was also strongest at baseline; however, attenuation was statistically evident only after MPD, whereas PPD showed an intermediate pattern. Although circulating FGF21 concentrations increased significantly only after PPD, the FGF21–ferritin association after PPD did attenuated after MPD, despite no corresponding increase in circulating FGF21. This pattern suggests that circulating FGF21 concentrations and the strength of the FGF21–ferritin association may not change in parallel, potentially reflecting diet-specific differences in how FGF21 signaling relates to iron storage pathways. Overall, these findings support FGF21 as a related nutrient-responsive signal but provide stronger evidence for methionine as the primary predictor of ferritin handling in this study.

The results are compatible with a potential methionine-sensitive nutrient-sensing pathway linked to ferritin regulation, with FGF21 acting as a possible related or downstream signal. Experimental models have shown that amino acid–responsive signaling, including FGF21, activating transcription factor 4, and related stress-response pathways, can interact with iron metabolism and ferritin expression (53, 54). Preclinical research has also suggested a potential direct link between methionine metabolism and hepatic iron handling (55), though this relationship remains largely unexplored. As an exploratory secondary analysis, these findings should be considered hypothesis-generating warranting an investigation of the role of methionine-FGF21-ferritin pathway. Future mechanistic work is needed to determine whether methionine availability directly influences hepatic iron storage or whether the observed associations reflect broader adaptive responses to dietary protein quality and controlled diet exposure.

From a translational perspective, these findings support the potential role of pulse-based protein sources as affordable, shelf-stable, and accessible dietary protein options for older adults, particularly in rural communities or settings with limited year-round access to fresh foods (56, 57). This is consistent with USDA-based nutrient-density and cost analyses showing pulses rank among the lowest-cost protein sources per 100 g and per 100 kcal compared with animal proteins, with pulses costing approximately $0.36 per 100 g (57). Additionally, in our cohort, self-reported acceptability of pulses was high, further supported by strong adherence to the meals provided, minimal intake of non-study foods during the intervention, and minimal reported gastrointestinal discomfort (37). In this context, pulses may provide a practical strategy to improve dietary quality and diversify protein intake without compromising iron homeostasis.

This study has several strengths, including its randomized crossover controlled-feeding design, the use of DGA-aligned dietary patterns, and the side-by-side comparison of animal- and pulse-protein sources in rural older adults who were primarily unaccustomed to pulse-intake before enrolling in the study. The integration of iron-status biomarkers, sulfur amino acid-related metabolites, and nutrient-sensing hormones allowed a more comprehensive evaluation of iron homeostasis than intake data alone. However, several limitations should be noted. First, this was an exploratory secondary analysis of the parent PRODMED1 trial, which was not originally powered to test nutrient–iron biomarker associations, three-way interactions, or sex-stratified effects. Therefore, the observed methionine–FGF21–ferritin relationships should be interpreted as hypothesis-generating rather than causal. Second, although the crossover controlled-feeding design strengthens internal validity, the sample size was modest, particularly among men, limiting statistical power for sex-specific interpretation. Third, iron absorption was not directly measured; therefore, conclusions regarding dietary iron bioavailability are based on circulating biomarkers and dietary composition rather than direct absorption data. Relatedly, heme and non-heme iron were estimated rather than directly measured in foods. The iron compensation index was also used only as a simplified contextual tool and should not be interpreted as a validated or mechanistic predictor of iron absorption. In addition, MVPA findings were exploratory and should be confirmed in larger studies designed to test causal mechanisms. Finally, participants were free-living rural older adults, and findings may not generalize to younger adults, more diverse populations.

In conclusion, this exploratory analysis suggests that a DGA-aligned, PPD may be compatible with maintained iron homeostasis in older adults despite exclusive non-heme iron intake, though this interpretation should be confirmed in larger, adequately powered studies. These findings underscore the importance of overall diet quality, nutrient interactions, and physiological regulation in interpreting iron status. Methionine and FGF21were associated with ferritin, suggesting a potential methionine–FGF21–ferritin axis within a pulse-based dietary context that warrants further mechanistic investigation. Future studies should build on these exploratory findings through larger, adequately powered controlled-feeding trials designed specifically to test nutrient–iron biomarker relationships, sex-specific responses, and clinically relevant changes in iron status. Direct measures of iron absorption, together with direct chemical analysis of heme and non-heme iron content and key dietary modifiers such as phytate and vitamin C, would help validate the dietary matrix interpretation proposed here. Mechanistic studies are also needed to determine whether methionine availability directly influences ferritin regulation or whether this relationship reflects broader nutrient-sensing responses involving FGF21 and related hepatic pathways.

## Data Availability

The original contributions presented in the study are included in the article/Supplementary material, further inquiries can be directed to the corresponding author/s.
