# Supplementary material for: A DGA-aligned pulse-protein diet may support iron homeostasis in older adults: an exploratory analysis of the PRODMED1 trial
Source: Front Nutr. 2026 Aug 24;13:1889521. doi: 10.3389/fnut.2026.1889521 (PMC13547033; doi:10.3389/fnut.2026.1889521)
Supplement: Supplementary file 1 [file Supplementary_file_1.DOCX]

**Supplementary Material**

**Title:**

**A DGA-Aligned Pulse-protein Diet May Support Iron Homeostasis in Older Adults: An Exploratory Analysis of the PRODMED1 Trial**

**Bruna O. de Vargas** et al.

**Table S1.** Pearson’s correlation coefficients between serum biomarkers across dietary phases and sex.

| **Pearson's correlations: BSL all** | | | | | | | | | |
| --- | --- | --- | --- | --- | --- | --- | --- | --- | --- |
| **R value** | **Cystine** | **Methionine** | **logHomocysteine** | **logFolate** | **logVitaminB12** | **logFGF21** | **logIGF-1** | **IGFBP3** | **SAA** |
| **Iron** | -0.20 | 0.11 | 0.13 | -0.08 | -0.27 | -0.01 | 0.01 | -0.06 | -0.13 |
| **logTIBC** | 0.17 | 0.44^**^ | 0.26 | -0.08 | -0.20 | 0.04 | 0.00 | -0.15 | 0.36^*^ |
| **TSAT** | -0.21 | -0.03 | 0.03 | -0.03 | -0.18 | -0.03 | 0.02 | 0.00 | -0.20 |
| **logFerritin** | -0.12 | -0.01 | 0.01 | -0.25 | -0.06 | 0.28 | 0.07 | 0.10 | -0.12 |
| **logCRP** | 0.03 | -0.04 | -0.26 | -0.16 | 0.25 | -0.04 | 0.06 | 0.03 | 0.01 |
| **logHepcidin** | -0.31* | -0.23 | 0.00 | -0.07 | -0.05 | 0.05 | 0.09 | 0.09 | -0.39^**^ |
| **logErythroferrone** | 0.07 | 0.00 | -0.02 | 0.18 | -0.15 | 0.03 | -0.04 | 0.02 | 0.07 |
| **sTfR** | 0.25 | 0.39^**^ | -0.14 | 0.15 | 0.11 | 0.00 | 0.03 | -0.15 | 0.41^**^ |
| **logsTfR Index** | 0.23 | 0.31^*^ | -0.09 | 0.24 | 0.09 | -0.12 | -0.03 | -0.17 | 0.36^*^ |
| **Pearson's correlations: BSL men** | | | | | | | | | |
| **R value** | **Cystine** | **Methionine** | **Homocysteine** | **logFolate** | **logVitaminB12** | **logFGF21** | **IGF-1** | **IGFBP3** | **SAA** |
| **Iron** | -0.65^*^ | -0.17 | 0.38 | -0.23 | -0.44 | 0.27 | 0.31 | -0.07 | -0.68^*^ |
| **TIBC** | 0.05 | 0.67^*^ | 0.29 | 0.02 | -0.23 | 0.00 | 0.09 | -0.04 | 0.28 |
| **TSAT** | -0.51 | -0.34 | 0.23 | -0.16 | -0.25 | 0.23 | 0.25 | 0.00 | -0.60^*^ |
| **logFerritin** | -0.28 | -0.47 | -0.26 | -0.08 | 0.02 | 0.23 | 0.16 | 0.17 | -0.43 |
| **CRP** | 0.10 | -0.32 | -0.62^*^ | -0.07 | 0.79^**^ | -0.07 | -0.35 | -0.21 | -0.02 |
| **logHepcidin** | -0.64^*^ | -0.08 | -0.04 | -0.08 | -0.33 | 0.17 | -0.08 | -0.07 | -0.64^*^ |
| **Erythroferrone** | 0.01 | 0.06 | 0.08 | -0.30 | -0.38 | 0.12 | 0.62^*^ | 0.50 | 0.03 |
| **sTfR** | 0.48 | 0.25 | -0.24 | 0.35 | 0.39 | 0.04 | -0.01 | 0.10 | 0.54 |
| **logsTfR Index** | 0.43 | 0.49 | 0.06 | 0.27 | 0.18 | -0.04 | -0.14 | -0.07 | 0.58^*^ |
| **Pearson's correlations: BSL women** | | | | | | | | | |
| **R value** | **Cystine** | **Methionine** | **Homocysteine** | **logFolate** | **logVitaminB12** | **logFGF21** | **logIGF-1** | **IGFBP3** | **SAA** |
| **Iron** | 0.05 | 0.18 | -0.07 | 0.00 | -0.17 | -0.08 | -0.09 | -0.03 | 0.14 |
| **TIBC** | 0.17 | 0.09 | 0.13 | -0.12 | -0.10 | 0.19 | -0.02 | -0.09 | 0.20 |
| **TSAT** | 0.01 | 0.15 | -0.11 | 0.03 | -0.16 | -0.13 | -0.07 | -0.01 | 0.09 |
| **logFerritin** | 0.04 | 0.43^*^ | 0.23 | -0.39 | -0.19 | 0.32 | 0.06 | 0.03 | 0.25 |
| **logCRP** | -0.03 | 0.04 | -0.16 | -0.20 | 0.09 | -0.03 | 0.19 | 0.07 | -0.01 |
| **logHepcidin** | -0.02 | -0.18 | 0.14 | -0.10 | 0.07 | -0.03 | 0.21 | 0.10 | -0.11 |
| **logErythroferrone** | 0.07 | -0.05 | -0.09 | 0.32 | -0.06 | 0.02 | -0.17 | -0.06 | 0.04 |
| **sTfR** | 0.06 | 0.34 | -0.25 | 0.08 | 0.04 | 0.03 | 0.05 | -0.18 | 0.22 |
| **logsTfR Index** | 0.04 | 0.02 | -0.34^*^ | 0.30 | 0.14 | -0.14 | -0.01 | -0.15 | 0.05 |
| **Pearson's correlations: MPD all** | | | | | | | | | |
| **R value** | **logCystine** | **Methionine** | **Homocysteine** | **logFolate** | **logVitaminB12** | **logFGF21** | **IGF-1** | **logIGFBP3** | **SAA** |
| **Iron** | 0.13 | 0.14 | 0.09 | -0.05 | -0.07 | 0.10 | -0.10 | -0.13 | 0.20 |
| **logTIBC** | 0.07 | 0.30^*^ | 0.45^**^ | -0.01 | -0.17 | 0.20 | -0.07 | -0.28 | 0.16 |
| **TSAT** | 0.09 | 0.03 | -0.08 | -0.05 | -0.01 | -0.01 | -0.07 | -0.01 | 0.12 |
| **logFerritin** | 0.11 | 0.20 | 0.07 | -0.17 | -0.09 | 0.20 | 0.16 | 0.01 | 0.16 |
| **logCRP** | 0.08 | 0.04 | -0.13 | 0.00 | 0.28 | -0.03 | -0.07 | 0.07 | 0.10 |
| **logHepcidin** | -0.18 | -0.09 | -0.08 | 0.07 | -0.13 | -0.01 | -0.03 | 0.05 | -0.18 |
| **logErythroferrone** | 0.06 | -0.17 | -0.03 | 0.12 | -0.10 | -0.22 | -0.05 | 0.05 | -0.04 |
| **sTfR** | 0.12 | 0.04 | -0.13 | 0.04 | 0.03 | -0.06 | -0.16 | -0.18 | 0.11 |
| **logsTfR Index** | 0.03 | -0.04 | -0.09 | 0.09 | 0.02 | -0.16 | -0.21 | -0.16 | 0.01 |
| **Pearson's correlations: MPD men** | | | | | | | | | |
| **R value** | **logCystine** | **Methionine** | **Homocysteine** | **Folate** | **logVitaminB12** | **FGF21** | **IGF-1** | **logIGFBP3** | **SAA** |
| **Iron** | 0.23 | -0.06 | 0.42 | 0.18 | 0.10 | 0.14 | 0.17 | -0.11 | 0.22 |
| **TIBC** | -0.27 | 0.46 | 0.45 | 0.28 | -0.20 | -0.05 | -0.15 | -0.25 | -0.14 |
| **TSAT** | 0.32 | -0.23 | 0.20 | -0.01 | 0.16 | 0.12 | 0.19 | -0.01 | 0.27 |
| **Ferritin** | -0.10 | -0.37 | -0.04 | -0.08 | -0.13 | -0.04 | 0.60^*^ | 0.09 | -0.18 |
| **logCRP** | 0.07 | -0.28 | -0.67^*^ | -0.11 | 0.70^**^ | 0.13 | -0.38 | 0.35 | 0.00 |
| **Hepcidin** | -0.48 | -0.08 | 0.16 | 0.32 | -0.52 | -0.29 | 0.42 | -0.03 | -0.43 |
| **Erythroferrone** | -0.41 | 0.10 | -0.14 | 0.00 | -0.18 | -0.23 | 0.51 | 0.48 | -0.36 |
| **sTfR** | 0.42 | 0.03 | -0.19 | 0.06 | 0.23 | 0.30 | -0.29 | -0.03 | 0.36 |
| **sTfR Index** | 0.10 | 0.34 | 0.10 | -0.08 | -0.03 | -0.03 | -0.45 | -0.19 | 0.16 |
| **Pearson's correlations: MPD women** | | | | | | | | | |
| **R value** | **logCystine** | **Methionine** | **logHomocysteine** | **logFolate** | **logVitaminB12** | **logFGF21** | **IGF-1** | **IGFBP3** | **SAA** |
| **Iron** | 0.11 | 0.31 | 0.04 | -0.13 | -0.15 | -0.02 | -0.14 | -0.13 | 0.24 |
| **TIBC** | 0.28 | 0.17 | 0.50^**^ | -0.16 | -0.17 | 0.44^**^ | -0.11 | -0.30 | 0.33 |
| **TSAT** | 0.02 | 0.23 | -0.13 | -0.08 | -0.10 | -0.18 | -0.09 | 0.01 | 0.13 |
| **logFerritin** | 0.10 | 0.45^**^ | 0.17 | -0.22 | -0.16 | 0.27 | 0.02 | 0.02 | 0.23 |
| **logCRP** | 0.08 | 0.07 | 0.00 | 0.01 | 0.16 | 0.01 | -0.01 | 0.08 | 0.11 |
| **logHepcidin** | -0.04 | 0.09 | -0.02 | 0.10 | -0.01 | 0.10 | 0.01 | 0.14 | 0.01 |
| **logErythroferrone** | 0.17 | -0.25 | 0.06 | 0.14 | -0.07 | -0.19 | -0.21 | -0.02 | 0.03 |
| **sTfR** | -0.03 | -0.04 | -0.04 | -0.06 | -0.10 | -0.07 | -0.11 | -0.13 | -0.05 |
| **sTfR Index** | -0.02 | -0.33 | -0.05 | 0.04 | -0.05 | -0.22 | -0.06 | -0.10 | -0.14 |
| **Pearson's correlations: PPD all** | | | | | | | | | |
| **R value** | **logCystine** | **Methionine** | **Homocysteine** | **logFolate** | **logVitaminB12** | **logFGF21** | **IGF-1** | **IGFBP3** | **logSAA** |
| **Iron** | -0.05 | 0.35^*^ | -0.01 | -0.20 | -0.25 | -0.31^*^ | 0.00 | 0.18 | 0.07 |
| **logTIBC** | -0.03 | -0.06 | 0.46^**^ | -0.09 | -0.30^*^ | 0.09 | -0.10 | -0.06 | -0.06 |
| **TSAT** | -0.03 | 0.38^**^ | -0.18 | -0.16 | -0.10 | -0.32^*^ | 0.06 | 0.20 | 0.10 |
| **logFerritin** | 0.06 | -0.02 | -0.15 | -0.05 | 0.04 | 0.28 | 0.13 | 0.10 | 0.04 |
| **logCRP** | 0.14 | -0.12 | -0.17 | 0.10 | 0.30^*^ | 0.18 | 0.17 | 0.04 | 0.06 |
| **logHepcidin** | -0.16 | -0.26 | 0.13 | -0.14 | -0.06 | -0.14 | -0.11 | 0.00 | -0.23 |
| **logErythroferrone** | -0.14 | -0.02 | 0.00 | 0.13 | 0.01 | -0.32^*^ | 0.01 | -0.18 | -0.13 |
| **sTfR** | 0.13 | -0.26 | -0.29^*^ | 0.19 | 0.18 | 0.12 | -0.06 | -0.24 | 0.06 |
| **logsTfR Index** | 0.09 | -0.15 | -0.09 | 0.14 | 0.06 | -0.07 | -0.09 | -0.21 | 0.05 |
| **Pearson's correlations: PPD men** | | | | | | | | | |
| **R value** | **logCystine** | **Methionine** | **Homocysteine** | **logFolate** | **VitaminB12** | **FGF21** | **IGF-1** | **logIGFBP3** | **logSAA** |
| **Iron** | 0.20 | 0.44 | -0.08 | -0.02 | -0.05 | -0.26 | 0.29 | 0.36 | 0.30 |
| **TIBC** | -0.17 | -0.01 | 0.52 | 0.25 | -0.14 | 0.00 | -0.14 | -0.12 | -0.10 |
| **TSAT** | 0.30 | 0.40 | -0.21 | -0.17 | -0.01 | -0.17 | 0.28 | 0.39 | 0.35 |
| **Ferritin** | -0.16 | -0.05 | -0.13 | -0.15 | -0.16 | -0.06 | 0.45 | 0.21 | -0.15 |
| **logCRP** | 0.24 | -0.29 | -0.44 | -0.13 | 0.22 | -0.04 | -0.50 | -0.18 | 0.02 |
| **Hepcidin** | -0.36 | -0.61^*^ | -0.08 | -0.17 | -0.44 | -0.30 | 0.09 | -0.07 | -0.52 |
| **Erythroferrone** | -0.25 | -0.26 | -0.20 | -0.09 | -0.28 | -0.19 | 0.17 | -0.18 | -0.27 |
| **sTfR** | -0.18 | -0.47 | -0.37 | 0.21 | 0.06 | 0.36 | -0.12 | -0.35 | -0.32 |
| **logsTfR Index** | 0.11 | -0.12 | 0.32 | 0.10 | -0.06 | 0.11 | -0.37 | -0.32 | 0.04 |
| **Pearson's correlations: PPD women** | | | | | | | | | |
| **R value** | **logCystine** | **Methionine** | **Homocysteine** | **Folate** | **logVitaminB12** | **logFGF21** | **logIGF-1** | **IGFBP3** | **logSAA** |
| **Iron** | -0.16 | 0.34^*^ | 0.01 | -0.32 | -0.41^*^ | -0.38^*^ | -0.08 | 0.08 | -0.05 |
| **logTIBC** | 0.05 | -0.21 | 0.51^**^ | -0.32 | -0.36^*^ | 0.20 | -0.25 | -0.03 | -0.05 |
| **TSAT** | -0.19 | 0.43^*^ | -0.19 | -0.17 | -0.23 | -0.45^**^ | 0.03 | 0.08 | -0.04 |
| **logFerritin** | 0.17 | 0.10 | -0.07 | -0.01 | -0.01 | 0.41^*^ | 0.11 | 0.02 | 0.18 |
| **logCRP** | 0.12 | -0.14 | -0.10 | 0.11 | 0.35^*^ | 0.30 | 0.27 | 0.11 | 0.06 |
| **logHepcidin** | -0.11 | 0.11 | 0.20 | -0.07 | 0.00 | -0.05 | 0.02 | -0.08 | -0.09 |
| **logErythroferrone** | -0.09 | 0.10 | 0.06 | 0.20 | 0.06 | -0.34^*^ | -0.08 | -0.18 | -0.06 |
| **sTfR** | 0.24 | -0.28 | -0.30 | 0.20 | 0.23 | 0.03 | -0.15 | -0.18 | 0.19 |
| **logsTfR Index** | 0.14 | -0.25 | -0.18 | 0.13 | 0.16 | -0.15 | -0.15 | -0.12 | 0.10 |

Data are presented as Pearson’s correlation coefficients (R values). Non-normally distributed data were log-transformed, while normally distributed data were analyzed without transformation. ^*^p < 0.05; ^**^p < 0.01. Abbreviations: BSL, baseline; CRP, C-reactive protein; FGF21, fibroblast growth factor 21; IGF-1, insulin-like growth factor 1; IGFBP3, insulin-like growth factor binding protein 3; MPD, meat-protein diet; PPD, pulse-protein diet; sTfR, soluble transferrin receptor; sTfR Index, ratio of sTfR to log ferritin; SAA, total sulfur amino acids; TIBC, total iron-binding capacity; TSAT, transferrin saturation.

**Table S2.** Robust linear mixed model estimated slopes, 95% confidence intervals, and contrasts for ferritin.

| *model_all <- rlmer(****Ferritin*** *~ Methionine * Timepoint * Sex + logFGF21 * Timepoint * Sex + (1 \| ID))* | | | | | | |
| --- | --- | --- | --- | --- | --- | --- |
| A) **Overall slope of methionine and FGF21 across all IDs and diet phase** | | | | | | |
| **Methionine overall slope** | **Estimate** | **SE** | ***t* value** | **Lower CI (95%)** | **Upper CI (95%)** | ***p* value** |
| All | 15.2 | 3.6 | 4.2 | 8.03 | 22.3 | <0.0001 |
| **FGF21 overall slope** | **Estimate** | **SE** | ***t* value** | **Lower CI (95%)** | **Upper CI (95%)** | ***p* value** |
| All | 26.2 | 10.5 | 2.5 | 5.7 | 46.7 | 0.012 |
| B) **Pairwise contrast of methionine and FGF21 slopes by diet phase and all IDs** | | | | | | |
| **Pairwise contrast of Methionine slopes by diet phase (all IDs)** | | | | | | |
| **Contrast** | **Estimate** | **SE** | **Z ratio** | ***p* value** |  |  |
| BSL – Post MPD | 23.8 | 7.2 | 3.3 | 0.003 |  |  |
| BSL – Post PPD | 24.4 | 7.1 | 3.4 | 0.002 |  |  |
| Post MPD – Post PPD | 0.59 | 6.7 | 0.09 | 0.996 |  |  |
| **Pairwise contrast of FGF21 slopes by diet phase (all IDs)** | | | | | | |
| **Contrast** | **Estimate** | **SE** | **Z ratio** | ***p* value** |  |  |
| BSL – Post MPD | 45.3 | 17.1 | 2.6 | 0.023 |  |  |
| BSL – Post PPD | 30.9 | 15.8 | 1.96 | 0.123 |  |  |
| Post MPD – Post PPD | -14.4 | 14.9 | -0.96 | 0.600 |  |  |
| C) **Methionine and FGF21 slopes by diet phase and all IDs** | | | | | | |
| **Methionine slopes by diet phase (all IDs)** | | | | | | |
| **Timepoint** | **Estimate** | **SE** | ***t* value** | **Lower CI (95%)** | **Upper CI (95%)** | ***p* value** |
| BSL | 31.2 | 5.9 | 5.3 | 19.7 | 42.7 | <0.0001 |
| Post MPD | 7.4 | 6.0 | 1.2 | -4.3 | 19.2 | 0.216 |
| Post PPD | 6.8 | 4.3 | 1.6 | -1.5 | 15.2 | 0.109 |
| **FGF21 slopes by diet phase (all IDs)** | | | | | | |
| **Timepoint** | **Estimate** | **SE** | ***t* value** | **Lower CI (95%)** | **Upper CI (95%)** | ***p* value** |
| BSL | 51.6 | 15.9 | 3.2 | 20.4 | 82.7 | 0.001 |
| Post MPD | 6.3 | 13.7 | 0.46 | -20.6 | 33.2 | 0.647 |
| Post PPD | 20.7 | 11.9 | 1.7 | -2.7 | 44.1 | 0.083 |
| D) **Comparison of methionine and FGF21 slopes across diet phase within each sex** | | | | | | |
| **Methionine: Compare slopes across diet phase within each sex** | | | | | | |
| **Contrast** | **Estimate** | **SE** | **Z ratio** | ***p* value** |  |  |
| Sex = women |  |  |  |  |  |  |
| BSL – Post MPD | 6.418 | 6.17 | 1.040 | 0.551 |  |  |
| BSL – Post PPD | 7.462 | 7.49 | 0.996 | 0.579 |  |  |
| Post MPD – Post PPD | 1.044 | 6.53 | 0.160 | 0.986 |  |  |
| Sex = men |  |  |  |  |  |  |
| BSL – Post MPD | 41.209 | 13.0 | 3.162 | 0.004 |  |  |
| BSL – Post PPD | 41.334 | 12.1 | 3.418 | 0.002 |  |  |
| Post MPD – Post PPD | 0.125 | 11.6 | 0.011 | 0.999 |  |  |
| **FGF21: Compare slopes across diets within each sex** | | | | | | |
| **Contrast** | **Estimate** | **SE** | **Z ratio** | ***p* value** |  |  |
| Sex = women |  |  |  |  |  |  |
| BSL – Post MPD | -3.7 | 14.3 | -0.259 | 0.964 |  |  |
| BSL – Post PPD | -9.1 | 14.6 | -0.625 | 0.807 |  |  |
| Post MPD – Post PPD | -5.4 | 14.9 | -0.362 | 0.930 |  |  |
| Sex = men |  |  |  |  |  |  |
| BSL – Post MPD | 94.2 | 31.2 | 3.024 | 0.007 |  |  |
| BSL – Post PPD | 70.8 | 28.0 | 2.531 | 0.031 |  |  |
| Post MPD – Post PPD | -23.4 | 25.9 | -0.905 | 0.637 |  |  |
| E) **Comparison of methionine and FGF21 slopes between sexes at each diet phase** | | | | | | |
| **Methionine: Compare slopes between sexes at each diet phases** | | | | | | |
| **Timepoint** | **Estimate** | **SE** | **Z ratio** | ***p* value** |  |  |
| BSL women-men | -29.88 | 11.7 | -2.544 | 0.011 |  |  |
| Post MPD women-men | 4.91 | 12.0 | 0.409 | 0.683 |  |  |
| Post PPD women-men | 3.99 | 8.54 | 0.467 | 0.641 |  |  |
| **FGF21: Compare slopes between sexes at each diet phases** | | | | | | |
| **Timepoint** | **Estimate** | **SE** | **Z ratio** | ***p* value** |  |  |
| BSL women-men | -43.1 | 31.8 | -1.354 | 0.176 |  |  |
| Post MPD women-men | 54.9 | 27.4 | 2.000 | 0.046 |  |  |
| Post PPD women-men | 36.9 | 23.9 | 1.545 | 0.122 |  |  |
| F) **Methionine and FGF21 slopes by sex** | | | | | | |
| **Methionine slopes by sex** | | | | | | |
| **Timepoint** | **Estimate** | **SE** | ***t* value** | **Lower CI (95%)** | **Upper CI (95%)** | ***p* value** |
| BSL men | 46.2 | 10.5 | 4.4 | 25.6 | 66.8 | <0.0001 |
| BSL women | 16.3 | 5.3 | 3.1 | 6.0 | 26.6 | 0.002 |
| Post MPD men | 5.0 | 11.2 | 0.44 | -17.1 | 27.0 | 0.659 |
| Post MPD women | 9.9 | 4.2 | 2.4 | 1.7 | 18.1 | 0.018 |
| Post PPD men | 4.8 | 6.4 | 0.76 | -7.7 | 17.4 | 0.449 |
| Post PPD women | 8.8 | 5.7 | 1.6 | -2.3 | 19.9 | 0.118 |
| **FGF21 slopes by sex** | | | | | | |
| **Timepoint** | **Estimate** | **SE** | ***t* value** | **Lower CI (95%)** | **Upper CI (95%)** | ***p* value** |
| BSL men | 73.1 | 30.0 | 2.4 | 14.2 | 131.9 | 0.015 |
| BSL women | 30.0 | 10.5 | 2.9 | 9.5 | 50.5 | 0.004 |
| Post MPD men | -21.2 | 24.0 | -0.88 | -68.1 | 25.8 | 0.378 |
| Post MPD women | 33.7 | 13.4 | 2.5 | 7.5 | 59.9 | 0.012 |
| Post PPD men | 2.2 | 19.9 | 0.11 | -36.7 | 41.2 | 0.910 |
| Post PPD women | 39.1 | 13.2 | 3.0 | 13.2 | 65.1 | 0.003 |

Abbreviations: BSL, baseline; FGF21, fibroblast growth factor 21; MPD, meat-protein diet; PPD, pulse-protein diet; SE, standard error.

**Table S3.** Robust linear mixed model estimated slopes, 95% confidence intervals, and contrasts for TIBC.

| *model_all <- rlmer(****TIBC*** *~ Methionine * Timepoint * Sex + logFGF21 * Timepoint * Sex + (1 \| ID))* | | | | | | |
| --- | --- | --- | --- | --- | --- | --- |
| A) **Overall slope of methionine and FGF21 across all IDs and diet phase** | | | | | | |
| **Methionine overall slope** | **Estimate** | **SE** | ***t* value** | **Lower CI (95%)** | **Upper CI (95%)** | ***p* value** |
| All | 4.7 | 5.6 | 0.84 | -6.2 | 15.6 | 0.399 |
| **FGF21 overall slope** | **Estimate** | **SE** | ***t* value** | **Lower CI (95%)** | **Upper CI (95%)** | ***p* value** |
| All | 17.6 | 15.1 | 1.2 | -11.9 | 47.1 | 0.241 |
| B) **Pairwise contrast of methionine and FGF21 slopes by diet phase and all IDs** | | | | | | |
| **Pairwise contrast of Methionine slopes by diet phase (all IDs)** | | | | | | |
| **Contrast** | **Estimate** | **SE**^1^ | **Z ratio** | ***p* value** |  |  |
| BSL– Post MPD | 10.33 | 12.1 | 0.850 | 0.672 |  |  |
| BSL – Post PPD | 20.16 | 11.8 | 1.705 | 0.203 |  |  |
| Post MPD – Post PPD | 9.83 | 11.0 | 0.895 | 0.643 |  |  |
| **Pairwise contrast of FGF21 slopes by diet phases (all IDs)** | | | | | | |
| **Contrast** | **Estimate** | **SE** | **Z ratio** | ***p* value** |  |  |
| BSL – Post MPD | -78.7 | 29.0 | -2.711 | 0.018 |  |  |
| BSL – Post PPD | -31.3 | 26.8 | -1.169 | 0.472 |  |  |
| Post MPD – Post PPD | 47.4 | 25.4 | 1.870 | 0.148 |  |  |
| C) **Methionine and FGF21 slopes by diet phase and all IDs** | | | | | | |
| **Methionine slopes by diet phase (all IDs)** | | | | | | |
| **Timepoint** | **Estimate** | **SE** | ***t* value** | **Lower CI (95%)** | **Upper CI (95%)** | ***p* value** |
| BSL | 14.9 | 9.6 | 1.5 | -4.0 | 33.7 | 0.122 |
| Post MPD | 4.5 | 9.3 | 0.5 | -13.8 | 22.8 | 0.627 |
| Post PPD | -5.3 | 7.0 | -0.8 | -19.1 | 8.5 | 0.452 |
| **FGF21 slopes by diet phase (all IDs)** | | | | | | |
| **Timepoint** | **Estimate** | **SE** | ***t* value** | **Lower CI (95%)** | **Upper CI (95%)** | ***p* value** |
| BSL | -19.1 | 24.5 | -0.78 | -67.1 | 28.9 | 0.436 |
| Post MPD | 59.7 | 21.8 | 2.73 | 16.9 | 102.5 | 0.006 |
| Post PPD | 12.2 | 18.4 | 0.67 | -23.8 | 48.3 | 0.506 |
| D) **Comparison of methionine and FGF21 slopes across diet phase within each sex** | | | | | | |
| **Methionine: Compare slopes across diets within each sex** | | | | | | |
| **Contrast** | **Estimate** | **SE** | **Z ratio** | ***p* value** |  |  |
| Sex = women |  |  |  |  |  |  |
| BSL – Post MPD | 9.32 | 10.5 | 0.887 | 0.649 |  |  |
| BSL – Post PPD | 14.89 | 12.6 | 1.183 | 0.463 |  |  |
| Post MPD – Post PPD | 5.57 | 11.1 | 0.501 | 0.871 |  |  |
| Sex = men |  |  |  |  |  |  |
| BSL – Post MPD | 11.34 | 21.9 | 0.518 | 0.863 |  |  |
| BSL – Post PPD | 25.43 | 20.0 | 1.270 | 0.412 |  |  |
| Post MPD – Post PPD | 14.09 | 18.9 | 0.744 | 0.737 |  |  |
| **FGF21: Compare slopes across diets within each sex** | | | | | | |
| **Contrast** | **Estimate** | **SE** | **Z ratio** | ***p* value** |  |  |
| Sex = men |  |  |  |  |  |  |
| BSL – Post MPD | -33.77 | 24.4 | -1.384 | 0.349 |  |  |
| BSL – Post PPD | -5.94 | 24.7 | -0.241 | 0.968 |  |  |
| Post MPD – Post PPD | 27.83 | 25.8 | 1.081 | 0.526 |  |  |
| Sex = men |  |  |  |  |  |  |
| BSL – Post MPD | -123.72 | 52.7 | -2.347 | 0.049 |  |  |
| BSL – Post PPD | -56.71 | 47.6 | -1.192 | 0.458 |  |  |
| Post MPD – Post PPD | 67.01 | 43.7 | 1.534 | 0.275 |  |  |
| E) **Comparison of methionine and FGF21 slopes between sexes at each diet phase** | | | | | | |
| **Methionine: Compare slopes between sexes at each timepoint** | | | | | | |
| **Timepoint** | **Estimate** | **SE** | **Z ratio** | ***p* value** |  |  |
| BSL women-men | -14.62 | 19.2 | -0.760 | 0.447 |  |  |
| Post MPD women-men | -12.60 | 18.7 | -0.675 | 0.500 |  |  |
| Post PPD women-men | -4.08 | 14.1 | -0.290 | 0.772 |  |  |
| **FGF21: Compare slopes between sexes at each timepoint** | | | | | | |
| **Timepoint** | **Estimate** | **SE** | **Z ratio** | ***p* value** |  |  |
| BSL women-men | 56.81 | 49.0 | 1.160 | 0.246 |  |  |
| Post MPD women-men | -33.15 | 43.7 | -0.759 | 0.448 |  |  |
| Post PPD women-men | 6.03 | 36.8 | 0.164 | 0.870 |  |  |
| F) **Methionine and FGF21 slopes by sex** | | | | | | |
| **Methionine slopes by sex** | | | | | | |
| **Timepoint** | **Estimate** | **SE** | ***t* value** | **Lower CI (95%)** | **Upper CI (95%)** | ***p* value** |
| BSL men | 22.2 | 17.1 | 1.3 | -11.4 | 55.7 | 0.195 |
| BSL women | 7.55 | 8.76 | 0.86 | -9.6 | 24.7 | 0.388 |
| Post MPD men | 10.8 | 17.3 | 0.63 | -23.1 | 44.8 | 0.532 |
| Post MPD women | -1.77 | 6.95 | -0.25 | -15.4 | 11.9 | 0.799 |
| Post PPD men | -3.3 | 10.5 | -0.31 | -23.8 | 17.3 | 0.757 |
| Post PPD women | -7.33 | 9.4 | -0.78 | -25.8 | 11.1 | 0.436 |
| **FGF21 slopes by sex** | | | | | | |
| **Timepoint** | **Estimate** | **SE** | ***t* value** | **Lower CI (95%)** | **Upper CI (95%)** | ***p* value** |
| BSL men | -47.5 | 46.0 | -1.03 | -137.6 | 42.6 | 0.302 |
| BSL women | 9.3 | 16.9 | 0.55 | -23.8 | 42.5 | 0.581 |
| Post MPD men | 76.2 | 38.1 | 2.00 | 1.51 | 151.0 | 0.046 |
| Post MPD women | 43.1 | 21.3 | 2.03 | 1.4 | 84.8 | 0.043 |
| Post PPD men | 9.2 | 30.1 | 0.31 | -49.8 | 68.3 | 0.759 |
| Post PPD women | 15.3 | 21.1 | 0.72 | -26.1 | 56.6 | 0.470 |

Abbreviations: BSL, baseline; FGF21, fibroblast growth factor 21; MPD, meat-protein diet; PPD, pulse-protein diet; SE, standard error; TIBC, total iron-binding capacity.

**Table S4.** Robust linear mixed model estimated slopes, 95% confidence intervals, and contrasts for TSAT.

| *model_all <- rlmer(****TSAT*** *~ Methionine * Timepoint * Sex + logFGF21 * Timepoint * Sex + (1 \| ID))* | | | | | | |
| --- | --- | --- | --- | --- | --- | --- |
| A) **Overall slope of methionine and FGF21 across all IDs and diet phase** | | | | | | |
| **Methionine overall slope** | **Estimate** | **SE** | ***t* value** | **Lower CI (95%)** | **Upper CI (95%)** | ***p* value** |
| All | -0.16 | 0.84 | -0.19 | -1.80 | 1.5 | 0.846 |
| **FGF21 overall slope** | **Estimate** | **SE** | ***t* value** | **Lower CI (95%)** | **Upper CI (95%)** | ***p* value** |
| All | -3.31 | 2.2 | -1.49 | -7.67 | 1.0 | 0.136 |
| B) **Pairwise contrast of methionine and FGF21 slopes by diet phase and all IDs** | | | | | | |
| **Pairwise contrast of Methionine slopes by diet phase (all IDs)** | | | | | | |
| **Contrast** | **Estimate** | **SE** | **Z ratio** | ***p* value** |  |  |
| BSL– Post MPD | -1.39 | 1.87 | -0.743 | 0.738 |  |  |
| BSL – Post PPD | -3.95 | 1.81 | -2.182 | 0.074 |  |  |
| Post MPD – Post PPD | -2.56 | 1.68 | -1.522 | 0.281 |  |  |
| **Pairwise contrast of FGF21 slopes by diet phase (all IDs)** | | | | | | |
| **Contrast** | **Estimate** | **SE** | **Z ratio** | ***p* value** |  |  |
| BSL – Post MPD | 2.88 | 4.48 | 0.642 | 0.797 |  |  |
| BSL – Post PPD | 8.75 | 4.14 | 2.116 | 0.087 |  |  |
| Post MPD – Post PPD | 5.87 | 3.91 | 1.501 | 0.291 |  |  |
| C) **Methionine and FGF21 slopes by diet phase and all IDs** | | | | | | |
| **Methionine slopes by diet phase (all IDs)** | | | | | | |
| **Timepoint** | **Estimate** | **SE** | ***t* value** | **Lower CI (95%)** | **Upper CI (95%)** | ***p* value** |
| BSL | -1.94 | 1.5 | -1.3 | -4.8 | 0.9 | 0.186 |
| Post MPD | -0.55 | 1.4 | -0.4 | -3.3 | 2.2 | 0.695 |
| Post PPD | 2.01 | 1.1 | 1.9 | -0.1 | 4.1 | 0.063 |
| **FGF21 slopes by diet phase (all IDs)** | | | | | | |
| **Timepoint** | **Estimate** | **SE** | ***t* value** | **Lower CI (95%)** | **Upper CI (95%)** | ***p* value** |
| BSL | 0.56 | 3.7 | 0.2 | -6.7 | 7.8 | 0.879 |
| Post MPD | -2.32 | 3.3 | -0.7 | -8.8 | 4.2 | 0.484 |
| Post PPD | -8.19 | 2.8 | -3.0 | -13.6 | -2.8 | 0.003 |
| D) **Comparison of methionine and FGF21 slopes across diet phase within each sex** | | | | | | |
| **Methionine: Compare slopes across diets within each sex** | | | | | | |
| **Contrast** | **Estimate** | **SE** | **Z ratio** | ***p* value** |  |  |
| Sex = women |  |  |  |  |  |  |
| BSL – Post MPD | -1.27 | 1.62 | -0.783 | 0.714 |  |  |
| BSL – Post PPD | -2.62 | 1.93 | -1.353 | 0.366 |  |  |
| Post MPD – Post PPD | -1.35 | 1.71 | -0.786 | 0.712 |  |  |
| Sex = men |  |  |  |  |  |  |
| BSL – Post MPD | -1.51 | 3.37 | -0.448 | 0.895 |  |  |
| BSL – Post PPD | -5.28 | 3.06 | -1.726 | 0.195 |  |  |
| Post MPD – Post PPD | -3.77 | 2.90 | -1.303 | 0.393 |  |  |
| **FGF21: Compare slopes across diets within each sex** | | | | | | |
| **Contrast** | **Estimate** | **SE** | **Z ratio** | ***p* value** |  |  |
| Sex = women |  |  |  |  |  |  |
| BSL – Post MPD | 2.53 | 3.77 | 0.678 | 0.776 |  |  |
| BSL – Post PPD | 7.59 | 3.80 | 1.995 | 0.113 |  |  |
| Post MPD – Post PPD | 5.03 | 3.99 | 1.260 | 0.418 |  |  |
| Sex = men |  |  |  |  |  |  |
| BSL – Post MPD | 3.20 | 8.13 | 0.394 | 0.918 |  |  |
| BSL – Post PPD | 9.91 | 7.34 | 1.350 | 0.368 |  |  |
| Post MPD – Post PPD | 6.71 | 6.73 | 0.997 | 0.579 |  |  |
| E) **Comparison of methionine and FGF21 slopes between sexes at each diet phase** | | | | | | |
| **Methionine: Compare slopes between sexes at each timepoint** | | | | | | |
| **Timepoint** | **Estimate** | **SE** | **Z ratio** | ***p* value** |  |  |
| BSL women-men | 3.13 | 2.9 | 1.07 | 0.287 |  |  |
| Post MPD women-men | 2.88 | 2.82 | 1.02 | 0.306 |  |  |
| Post PPD women-men | 0.46 | 2.16 | 0.21 | 0.831 |  |  |
| **FGF21: Compare slopes between sexes at each timepoint** | | | | | | |
| **Timepoint** | **Estimate** | **SE** | **Z ratio** | ***p* value** |  |  |
| BSL women-men | -1.510 | 7.4 | -0.204 | 0.838 |  |  |
| Post MPD women-men | -0.860 | 6.62 | -0.130 | 0.897 |  |  |
| Post PPD women-men | 0.819 | 5.55 | 0.148 | 0.883 |  |  |
| F) **Methionine and FGF21 slopes by sex** | | | | | | |
| **Methionine slopes by sex** | | | | | | |
| **Timepoint** | **Estimate** | **SE** | ***t* value** | **Lower CI (95%)** | **Upper CI (95%)** | ***p* value** |
| BSL men | -3.51 | 2.6 | -1.3 | -8.6 | 1.6 | 0.179 |
| BSL women | -0.38 | 1.3 | -0.3 | -3.0 | 2.25 | 0.778 |
| Post MPD men | -1.99 | 2.6 | -0.76 | -7.1 | 3.1 | 0.445 |
| Post MPD women | 0.89 | 1.1 | 0.84 | -1.2 | 2.98 | 0.402 |
| Post PPD men | 1.78 | 1.6 | 1.11 | -1.37 | 4.92 | 0.268 |
| Post PPD women | 2.24 | 1.4 | 1.55 | -0.59 | 5.1 | 0.121 |
| **FGF21 slopes by sex** | | | | | | |
| **Timepoint** | **Estimate** | **SE** | ***t* value** | **Lower CI (95%)** | **Upper CI (95%)** | ***p* value** |
| BSL men | 1.31 | 6.92 | 0.190 | -12.25 | 14.9 | 0.849 |
| BSL women | -0.19 | 2.57 | -0.08 | -5.24 | 4.85 | 0.940 |
| Post MPD men | -1.89 | 5.8 | -0.33 | -13.2 | 9.45 | 0.744 |
| Post MPD women | -2.75 | 3.22 | -0.85 | -9.06 | 3.57 | 0.394 |
| Post PPD men | -8.6 | 4.5 | -1.9 | -17.5 | 0.28 | 0.058 |
| Post PPD women | -7.8 | 3.2 | -2.4 | -14.05 | -1.5 | 0.015 |

Abbreviations: BSL, baseline; FGF21, fibroblast growth factor 21; MPD, meat-protein diet; PPD, pulse-protein diet; SE, standard error; TSAT, transferrin saturation.

**Table S5.** Robust linear mixed model estimated slopes, 95% confidence intervals, and contrasts for sTfR.

| *model_all <- rlmer(****sTfR*** *~ Methionine * Timepoint * Sex + logFGF21 * Timepoint * Sex + (1 \| ID))* | | | | | | |
| --- | --- | --- | --- | --- | --- | --- |
| A) **Overall slope of methionine and FGF21 across all IDs and diet phase** | | | | | | |
| **Methionine overall slope** | **Estimate** | **SE** | ***t* value** | **Lower CI (95%)** | **Upper CI (95%)** | ***p* value** |
| All | 0.039 | 0.09 | 0.45 | -0.14 | 0.21 | 0.656 |
| **FGF21 overall slope** | **Estimate** | **SE** | ***t* value** | **Lower CI (95%)** | **Upper CI (95%)** | ***p* value** |
| All | 0.32 | 0.24 | 1.33 | -0.15 | 0.80 | 0.184 |
| B) **Pairwise contrast of methionine and FGF21 slopes by diet phase and all IDs** | | | | | | |
| **Pairwise contrast of Methionine slopes by diet phase (all IDs)** | | | | | | |
| **Contrast** | **Estimate** | **SE** | **Z ratio** | ***p* value** |  |  |
| BSL– Post MPD | 0.178 | 0.185 | 0.961 | 0.601 |  |  |
| BSL – Post PPD | 0.376 | 0.182 | 2.066 | 0.097 |  |  |
| MPD - PPD | 0.197 | 0.169 | 1.168 | 0.472 |  |  |
| **Pairwise contrast of FGF21 slopes by diet phase (all IDs)** | | | | | | |
| **Contrast** | **Estimate** | **SE** | **Z ratio** | ***p* value** |  |  |
| BSL – Post MPD | 0.086 | 0.443 | 0.194 | 0.980 |  |  |
| BSL – Post PPD | 0.177 | 0.408 | 0.433 | 0.902 |  |  |
| MPD - PPD | 0.091 | 0.386 | 0.236 | 0.970 |  |  |
| C) **Methionine and FGF21 slopes by diet phase and all IDs** | | | | | | |
| **Methionine slopes by timepoint (all IDs)** | | | | | | |
| **Timepoint** | **Estimate** | **SE** | ***t* value** | **Lower CI (95%)** | **Upper CI (95%)** | ***p* value** |
| BSL | 0.22 | 0.15 | 1.51 | -0.07 | 0.52 | 0.132 |
| Post MPD | 0.05 | 0.15 | 0.31 | -0.24 | 0.33 | 0.755 |
| Post PPD | -0.15 | 0.11 | -1.40 | -0.36 | 0.06 | 0.163 |
| **FGF21 slopes by timepoint (all IDs)** | | | | | | |
| **Timepoint** | **Estimate** | **SE** | ***t* value** | **Lower CI (95%)** | **Upper CI (95%)** | ***p* value** |
| BSL | 0.41 | 0.39 | 1.07 | -0.35 | 1.17 | 0.287 |
| Post MPD | 0.33 | 0.34 | 0.96 | -0.34 | 0.99 | 0.339 |
| Post PPD | 0.23 | 0.29 | 0.81 | -0.33 | 0.80 | 0.418 |
| D) **Comparison of methionine and FGF21 slopes across diet phase within each sex** | | | | | | |
| **Methionine: Compare slopes across diets within each sex** | | | | | | |
| **Contrast** | **Estimate** | **SE** | **Z ratio** | ***p* value** |  |  |
| Sex = women |  |  |  |  |  |  |
| BSL – Post MPD | 0.246 | 0.160 | 1.537 | 0.273 |  |  |
| BSL – Post PPD | 0.370 | 0.193 | 1.921 | 0.133 |  |  |
| Post MPD – Post PPD | 0.124 | 0.169 | 0.734 | 0.743 |  |  |
| Sex = men |  |  |  |  |  |  |
| BSL – Post MPD | 0.111 | 0.335 | 0.330 | 0.942 |  |  |
| BSL – Post PPD | 0.381 | 0.308 | 1.236 | 0.432 |  |  |
| Post MPD – Post PPD | 0.270 | 0.292 | 0.925 | 0.624 |  |  |
| **FGF21: Compare slopes across diets within each sex** | | | | | | |
| **Contrast** | **Estimate** | **SE** | **Z ratio** | ***p* value** |  |  |
| Sex = women |  |  |  |  |  |  |
| BSL – Post MPD | -0.158 | 0.371 | -0.426 | 0.905 |  |  |
| BSL – Post PPD | -0.237 | 0.376 | -0.630 | 0.804 |  |  |
| Post MPD – Post PPD | -0.079 | 0.390 | -0.202 | 0.977 |  |  |
| Sex = men |  |  |  |  |  |  |
| BSL – Post MPD | 0.329 | 0.803 | 0.411 | 0.911 |  |  |
| BSL – Post PPD | 0.590 | 0.724 | 0.816 | 0.693 |  |  |
| Post MPD – Post PPD | 0.261 | 0.667 | 0.391 | 0.919 |  |  |
| E) **Comparison of methionine and FGF21 slopes between sexes at each diet phase** | | | | | | |
| **Methionine: Compare slopes between sexes at each timepoint** | | | | | | |
| **Timepoint** | **Estimate** | **SE** | **Z ratio** | ***p* value** |  |  |
| BSL women-men | -0.003 | 0.297 | -0.010 | 0.992 |  |  |
| Post MPD women-men | -0.138 | 0.293 | -0.472 | 0.637 |  |  |
| Post PPD women-men | 0.008 | 0.217 | 0.037 | 0.971 |  |  |
| **FGF21: Compare slopes between sexes at each timepoint** | | | | | | |
| **Timepoint** | **Estimate** | **SE** | **Z ratio** | ***p* value** |  |  |
| BSL women-men | -0.671 | 0.772 | -0.870 | 0.384 |  |  |
| Post MPD women-men | -0.183 | 0.681 | -0.269 | 0.787 |  |  |
| Post PPD women-men | 0.156 | 0.580 | 0.269 | 0.787 |  |  |
| F) **Methionine and FGF21 slopes by sex** | | | | | | |
| **Methionine slopes by sex** | | | | | | |
| **Timepoint** | **Estimate** | **SE** | ***t* value** | **Lower CI (95%)** | **Upper CI (95%)** | ***p* value** |
| BSL men | 0.225 | 0.26 | 0.85 | -0.29 | 0.745 | 0.395 |
| BSL women | 0.223 | 0.13 | 1.65 | -0.04 | 0.486 | 0.098 |
| MPD men | 0.115 | 0.27 | 0.42 | -0.42 | 0.650 | 0.674 |
| MPD women | -0.023 | 0.11 | -0.22 | -0.23 | 0.186 | 0.827 |
| PPD men | -0.156 | 0.16 | -0.96 | -0.47 | 0.162 | 0.337 |
| PPD women | -0.148 | 0.14 | -1.02 | -0.43 | 0.136 | 0.308 |
| **FGF21 slopes by sex** | | | | | | |
| **Timepoint** | **Estimate** | **SE** | ***t* value** | **Lower CI (95%)** | **Upper CI (95%)** | ***p* value** |
| BSL men | 0.75 | 0.73 | 1.029 | -0.675 | 2.169 | 0.303 |
| BSL women | 0.08 | 0.26 | 0.287 | -0.439 | 0.590 | 0.774 |
| MPD men | 0.417 | 0.59 | 0.701 | -0.748 | 1.58 | 0.483 |
| MPD women | 0.234 | 0.33 | 0.704 | -0.4^1^7 | 0.884 | 0.481 |
| PPD men | 0.156 | 0.477 | 0.327 | -0.779 | 1.092 | 0.743 |
| PPD women | 0.312 | 0.33 | 0.949 | 0.333 | 0.958 | 0.343 |

Abbreviations: BSL, baseline; FGF21, fibroblast growth factor 21; MPD, meat-protein diet; PPD, pulse-protein diet; SE, standard error; sTfR, soluble transferrin receptor.

**Table S6.** Overall slope tests for methionine and log-transformed FGF21 across iron-related outcomes with BH-FDR correction.

| **Outcome** | **Predictor** | **Estimate** | **SE** | **z value** | **95% CI** | ***p* value** | ***p* adj** |
| --- | --- | --- | --- | --- | --- | --- | --- |
| Ferritin | Methionine | 15.16 | 3.64 | 4.17 | 8.03, 22.30 | 0.00003 | 0.0002 |
| Ferritin | logFGF21 | 26.18 | 10.46 | 2.50 | 5.67, 46.68 | 0.012 | 0.049 |
| TSAT | Methionine | -0.16 | 0.84 | -0.19 | -1.80, 1.48 | 0.846 | 0.846 |
| TSAT | logFGF21 | -3.31 | 2.22 | -1.49 | -7.67, 1.04 | 0.136 | 0.361 |
| TIBC | Methionine | 4.70 | 5.58 | 0.84 | -6.23, 15.64 | 0.399 | 0.533 |
| TIBC | logFGF21 | 17.61 | 15.05 | 1.17 | -11.89, 47.12 | 0.242 | 0.387 |
| sTfR | Methionine | 0.04 | 0.09 | 0.45 | -0.13, 0.21 | 0.656 | 0.749 |
| sTfR | logFGF21 | 0.32 | 0.24 | 1.33 | -0.15, 0.80 | 0.184 | 0.369 |

Values represent overall slope estimates from MVPA linear mixed-effects models evaluating associations between methionine or log-transformed FGF21 and iron-related outcomes across all timepoints and participants. Models included timepoint, sex, nutrient-responsive predictor, and interaction terms, with participant ID included as a random effect. The 95% confidence intervals are shown for each estimate. Raw p-values and Benjamini-Hochberg false discovery rate-adjusted p-values are reported. Abbreviations: BH-FDR, Benjamini-Hochberg false discovery rate; FGF21, fibroblast growth factor 21; CI, confidence interval; TSAT, transferrin saturation; TIBC, total iron-binding capacity; sTfR, soluble transferrin receptor.

**Table S7.** Multicollinearity and random-effect variance components for MVPA models

| **Outcome** | **Maximum adjusted GVIF** | **Random intercept variance** | **Random intercept SD** | **Residual variance** | **Residual SD** |
| --- | --- | --- | --- | --- | --- |
| Ferritin | 2.78 | 2625.01 | 51.23 | 237.21 | 15.40 |
| TIBC | 2.78 | 907.57 | 30.13 | 729.50 | 27.01 |
| TSAT | 2.78 | 15.15 | 3.89 | 17.73 | 4.21 |
| sTfR | 2.78 | 0.36 | 0.60 | 0.16 | 0.41 |

Maximum adjusted GVIF values represent the highest GVIF^(1/[2×Df]) observed among fixed-effect terms within each outcome model. Random intercept variance and SD represent between-participant variability accounted for by participant ID, whereas residual variance and SD represent unexplained within-model variability. Abbreviations: GVIF, generalized variance inflation factor; SD, standard deviation; MVPA, multivariable predictive analysis; TSAT, transferrin saturation; TIBC, total iron-binding capacity; sTfR, soluble transferrin receptor.

**Figure S1. Residual diagnostic plots for MVPA models of iron-related outcomes.**  For each outcome, Q-Q plots of standardized residuals are shown in the left column and residuals-versus-fitted plots are shown in the right column. Outcomes include ferritin, sTfR, TSAT, and TIBC. Q-Q plots were used to assess residual distribution, while residuals-versus-fitted plots were used to evaluate potential systematic patterns across fitted values. Overall, residuals were generally centered around zero, with some tail deviations observed in the Q-Q plots. Abbreviations: MVPA, multivariable predictive analysis; Q-Q, quantile-quantile; TSAT, transferrin saturation; TIBC, total iron-binding capacity; sTfR, soluble transferrin receptor.
